# CRISPR/Cas9 Eye Drop HSV-1 Treatment Reduces Brain Viral Load: A Novel Application to Prevent Neuronal Damage

**DOI:** 10.3390/pathogens13121087

**Published:** 2024-12-10

**Authors:** Rafaela Moraes Pereira de Sousa, Luiza Silveira Garcia, Felipe Simões Lemos, Viviane Souza de Campos, Erik Machado Ferreira, Nathália Alves Araujo de Almeida, Tatiana Maron-Gutierrez, Elen Mello de Souza, Vanessa Salete de Paula

**Affiliations:** 1 Laboratório de Virologia e Parasitologia Molecular, Instituto Oswaldo Cruz/FIOCRUZ, Rio de Janeiro 21040900, RJ, Brazil; rafaelamoraesfiocruz@gmail.com (R.M.P.d.S.); luizagarcia@aluno.fiocruz.br (L.S.G.); natybio92@yahoo.com.br (N.A.A.d.A.); elenmellodesouza@gmail.com (E.M.d.S.); 2 Laboratório de Imunofarmacologia, Instituto Oswaldo Cruz/FIOCRUZ, Rio de Janeiro 21040-361, RJ, Brazil; simoeslemos.f@gmail.com (F.S.L.); tatiana.maron@fiocruz.br (T.M.-G.); 3 Laboratório de Biologia Estrutural, Instituto Oswaldo Cruz/FIOCRUZ, Rio de Janeiro 21040900, RJ, Brazil; vvncampos@gmail.com; 4 Laboratório de Hepatites Virais, Instituto Oswaldo Cruz/FIOCRUZ, Rio de Janeiro 21040900, RJ, Brazil; erikmafer@gmail.com

**Keywords:** viral infection, herpes simplex virus type 1, CRISPR/Cas9, neuronal damage, gene therapy

## Abstract

Herpes simplex virus-1 (HSV-1) can invade the central nervous system (CNS). However, antiviral drugs used to treat HSV-1 have significant toxicity and resistance. An alternative approach involves the use of the CRISPR/Cas9 complex as a viral replication inhibitor. Editing the *UL39* gene with CRISPR/Cas9 results in >95% inhibition of HSV-1 replication in vitro; however, few studies have investigated alternative therapies in in vivo models. This study aimed to investigate the efficacy of CRISPR/Cas9 targeting the *UL39* region, which was administered via the ocular route, to reduce the HSV-1 viral count in the CNS of BALB/c mice. Mice were inoculated with HSV-1 and treated using CRISPR/Cas9. The kinetics of CNS infection were assessed, and the effects of CRISPR/Cas9 were compared with those of topical acyclovir treatments. The brain viral load was analyzed, and histopathology and immunofluorescence of the nervous tissue were performed. The group treated with CRISPR/Cas9 showed a reduced viral load on the seventh day post-infection, and no brain inflammation or chromatin compaction was observed in animals that received CRISPR/Cas9 therapy. These findings suggest that CRISPR/Cas9 anti-*UL39* therapy can reduce the HSV-1 viral load in brain tissue. Therefore, investigating viral detection and evaluating antiviral treatments in the brain is essential.

## 1. Introduction

Simplex virus human alpha 1, or herpes simplex virus type 1 (HSV-1), is a double-stranded DNA virus of 152 kbp in size that belongs to the *Orthoherpesviridae* family and *Simplexvirus* genus [1]. Virus transmission occurs through direct exposure to HSV-1 in secretions from lesions or the saliva of infected individuals, even those who are asymptomatic [2]. HSV-1 typically infects the epithelial surfaces and establishes latency in sensory neurons [3]. The worldwide prevalence of HSV-1 infection in the general population (0–49 years) is approximately 67% [4]. However, HSV-1 prevalence exceeds 90% in Latin America and Sub-Saharan Africa [5]. After the primary infection, HSV-1 is transported by retrograde axonal transport to the neuronal cell bodies in the trigeminal ganglia, where latency is established [6,7]. HSV-1 can establish infections beyond the primary infection site through transport along sensory neurons. Herpetic infections can vary from asymptomatic or auto-limited to severe ocular and neurological disease [8,9,10].

Periodic reactivations result in recurrent clinical or subclinical episodes of disease throughout life [11]. Ocular infections can progress with viral reactivation in the peripheral ganglia and transmission to central nervous system (CNS) neurons [12]. The cornea is a highly innervated tissue that contains over 7000 nociceptors in humans [13], and virulent HSV-1 strains can infect the CNS [14]. Viral replication in the brain can cause acute necrotizing inflammation, known as herpes simplex encephalitis (HSE), which is typically localized in the temporal and sub-frontal areas [15]. This infection primarily affects immunocompromised individuals and newborns [16]. HSE leads to acute disease and inflammation associated with high morbidity and mortality [10,17], with an estimated global incidence of 2.5–12 cases/million per year [18,19,20]. The mortality rate is 70% in untreated patients and up to 30% in those treated with antivirals, with a high incidence of neurological injury [15].

The sequelae of HSV-1 infections in the CNS include impaired memory, psychiatric symptoms, anosmia, epilepsy, and dysphagia [15]. Recurrent symptoms occur in up to 10% of patients with HSE, even with negative cerebrospinal fluid PCR or low-level detection of HSV-1 DNA, indicating the necessity of antiviral therapy in recurrent cases [21]. Cumulative effects of repeated “mild” HSV-1 brain infections may cause neuronal damage similar to neurodegenerative diseases such as Alzheimer’s disease [22,23]. Therefore, treating CNS infections immediately is essential for patient recovery. The approved antiviral drugs against the virus are nucleoside analogues that block viral replication [24]. However, most of them target the viral DNA polymerase, which favors the emergence of resistant strains [25]. Acyclovir is the standard treatment for HSV-1 infections; however, a high rate of resistance has been reported, particularly in immunocompromised patients [26].

The evaluation of novel therapies against HSV-1 infection involves the knockdown of the UL39 region using siRNAs and the CRISPR system coupled with gene-specific endonuclease targeting. *UL39* and *UL40* encode the viral ribonucleotide reductase (RR) holoenzyme and convert diphosphates into triphosphates (dNTPs) that are used for DNA synthesis by cellular and viral DNA polymerase [27]. A mutation in the HSV-1 *UL39* gene attenuated the virus in vivo and was associated with a reduced level of antiapoptotic effects [28]. Nonsynonymous mutations in *UL39* can severely impair acute viral replication in the eyes and trigeminal ganglia of mice after corneal inoculation, affecting the establishment and reactivation of latency [28,29]. In 2020, Ebrahimi et al. constructed an oncolytic knockout of HSV-1 for the *UL39* region using the CRISPR/Cas9 system in HEK-293 cell culture, and replication was significantly impaired [30]. Thus, a mutation in *UL39* may reduce the deleterious effects of HSV-1 in CNS, which makes *UL39* a potential target for gene therapy. The CRISPR system works by directing guide RNA(s) (sgRNA) to target DNA sequences and with DNA cleavage enzymes (endonucleases) can silence the expression of a specific gene [31,32]. RR comprises two subunits encoded by *UL39* (large subunit) and *UL40* (small subunit) [33], and its activity is required, especially in non-replicating cells such as neurons, which have a reduced pool of dNTPs [34]. The use of CRISPR/Cas9 in vitro assays using the VERO cell line can inhibit HSV-1 replication at a rate of more than 95% [35].

Over the past 20 years, the *UL39* region of the HSV-1 genome has been targeted for silencing or mutation to inhibit HSV-1 replication. In 2008, Zhe et al. provided in vitro data demonstrating that silencing *UL39* expression using siRNAs resulted in inhibition rates ranging from 35% to 52% of HSV-1 replication in VERO cells [36]. Better results were achieved in 2011 using siRNAs targeting both *UL39* and *UL40*, achieving inhibition rates of 73% in vitro [37]. A study conducted in 2016 by da Silva et al. using siRNAs targeting the *UL39* region for the treatment of HSE successfully inhibited viral replication by 67.7% in the brains of BALB/c mice [38]. However, few studies have investigated alternative therapies for herpetic infections in in vivo models.

Therefore, in the present research, an in vivo model was conducted to assess the efficacy of topical CRISPR/Cas9 treatment targeting the *UL39* region in controlling ocular infections mimicking herpetic keratoconjunctivitis. Furthermore, considering the well-documented occurrence of CNS infection via the ocular route [39,40,41], this research also aimed to evaluate the reducing viral load in the CNS of mice. This study aimed to assess the efficacy of the CRISPR-Cas9 complex administered as ocular drops targeting the *UL39* gene of HSV-1 to mitigate the viral load within the CNS of HSV-1-infected BALB/c mice.

## 2. Materials and Methods

### 2.1. Virus and Cells

The Brazilian HSV-1 strain (GenBank accession number: JQ673480.1 NCBI: txid10306) was isolated from a human case of recurrent oral herpes with blisters and used for ocular infections in BALB/c mice. The African green monkey *Cercopithecus aethiops* kidney cell line (VERO cell line) was obtained from the Banco de Células do Rio de Janeiro (BCRJ), and cultures were used to propagate and determine viral titers [42]. Cells were maintained at 37 °C under a 5% CO_2_ atmosphere in a RPMI 1640 medium (Sigma-Aldrich, St. Louis, MO, USA) supplemented with 10% fetal bovine serum, 2% l-glutamine, and 1% penicillin/streptomycin. Viral stocks were stored at −80 °C.

### 2.2. Animals and Ethical Approval

The animals used in this study were sourced from the Institute of Science and Technology in Biomodels (ICTB/FIOCRUZ, Rio de Janeiro, Brazil). Five- to six-week-old male BALB/c mice, weighing between 20 and 25 g, were acclimatized for seven days before the experiment. The room temperature was 25 °C, and the relative humidity was 65%. The mice were maintained under an artificial 12:12 h light/dark cycle. Mouse chow and water were provided *ad libitum*. Five mice were housed in each cage and the assays were conducted in triplicate. Analyzes were made at three time points post-infection. All procedures adhered to the guidelines of the Ethics Committee on Animal Use at the Oswaldo Cruz Institute (approval no. CEUA-IOC L-35/2019) and the ARRIVE guidelines.

### 2.3. Experimental Ocular Infection

The animals were allocated to experimental groups (Table 1). Ocular infection was conducted in unscarred corneas by administering a 5 μL viral solution, which contained concentrations of 10^6^ or 10^9^ PFU/mL, which was injected directly into the right eyeball of the immobilized animal. During the same intervention, mice that were not infected (control group) received a 5 μL inoculum of phosphate-buffered saline (PBS) and were kept under identical conditions.

### 2.4. CRISPR/Cas9 and Pharmacological Treatment

The construction of CRISPR/Cas9-mediated genome editing targeting the *UL39* gene of HSV-1 and in vitro testing was previously detailed [35]. The present study used this approach to assess the therapeutic potential of CRISPR/Cas9 against HSV-1 infection in a CNS in vivo model. On the first day post-infection (dpi), each animal from the G4 group received a single application of 5 μL of CRISPR/Cas9 targeting anti-HSV-1, at a concentration of 200 ng/μL, directly into the right eyeball. The solution was prepared using CRISPR/Cas9 complex anti-UL39 diluted in PBS. The G5 group was treated with the standard drug acyclovir (150 mg/kg). Non-treated mice received a single application of 5 μL PBS and were kept under identical conditions.

**Figure 1:**
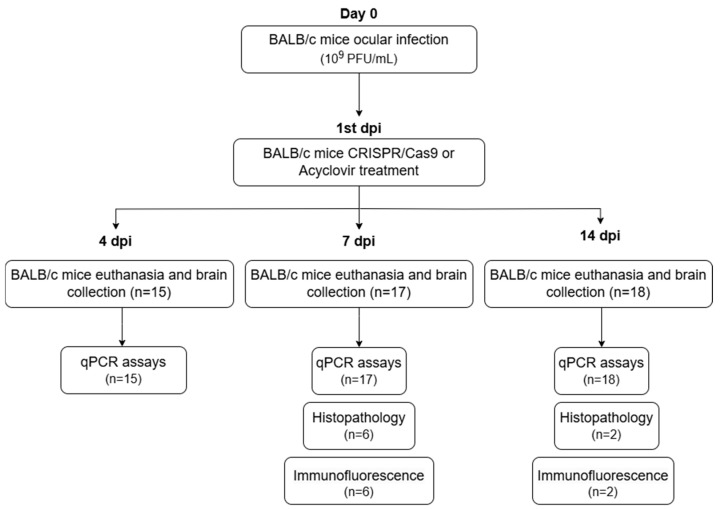
Flowchart of the infection, treatment, and sample collection stages. Dpi, days post-infection.

The mice were euthanized and dissected, and their brains were collected on the fourth, seventh, and fourteenth dpi. All collected samples were stored at −80 °C, except for the tissues selected for histopathological and immunofluorescence analyses, which were stored in buffered formalin and 4% paraformaldehyde, respectively. In total, 50 tissue samples were processed and tested (Figure 1).

### 2.5. DNA Extraction

HSV-1 DNA was extracted from all the collected tissues using a DNeasy Blood and Tissue Kit (QIAGEN, Valencia, CA, USA). All samples were macerated and sectioned into <25 mg tissue portions. DNA was extracted from the samples according to the manufacturer’s instructions.

### 2.6. Quantitative Real-Time PCR

Absolute quantification of HSV-1 DNA was performed using real-time qPCR (QuantStudio 3, Applied Biosystems, Waltham, MA, USA) [43]. The detection of HSV-1 in the brain tissue was measured through the identification of a 92-bp HSV-1 DNA fragment of *UL39* using the primers and probe targets in Table 2. The reaction was performed in a total volume of 13 μL using the AgPath-ID™ One-Step RT-PCR Kit (Life Technologies, Carlsbad, CA, USA). Each reaction consisted of 6 μL of 1× buffer (RT-PCR buffer), 1.25 μL of sense primers (1 μM), 1.25 μL of antisense primers (1 μM), 0.4 μL (0.4 μM) of the specific probe, and 0.5 μL of 1× enzyme per sample (3 μL). qPCR was also performed for negative controls, and known quantities of the HSV-1 DNA fragment were used to generate a standard curve.

### 2.7. Histopathology

Brain tissues were sent to the Histotechnology Platform at the Oswaldo Cruz Institute for the preparation of histological slides. The cerebral hemispheres were separated at collection: one hemisphere was designated for histopathological analysis, whereas the other was separated for molecular analysis. The tissue fragments were washed in PBS (10 mM sodium phosphate, 0.015 M NaCl, pH 7.4) and fixed in Millonig–Rosman solution (10% formaldehyde in phosphate buffer). The fixed tissues were processed in paraffin, and 3 µm sections were obtained. The slides were stained with hematoxylin and eosin (H and E).

### 2.8. Immunofluorescence

Paraffin-embedded brain slices were waxed three times in xylene for 10 min each and rehydrated in decreasing ethanol concentrations [44]. Slides were then heated at 95 °C in citrate buffer for 60 min. Primary antibodies against glial fibrillary acidic protein (GFAP; Invitrogen, Waltham, MA, USA) and HSV-1 (Bio-Rad, Hercules, CA, USA) were incubated overnight in Super Block (ScyTek, Logan, UT, USA). Secondary antibodies were applied for 2 h at 25 °C and conjugated with Alexa-488 and Alexa-546. Brain slices were covered with a mounting medium containing 4′,6-diamidino-2-phenylindole (DAPI) and covered with glass coverslips. Images were captured using an inverted microscope Axio Vert.A1 FL LED (Zeiss, Oberkochen, Germany) and processed using ImageJ software (v.1.54).

### 2.9. Statistical Analyses

Data obtained from three experiments were used for statistical analysis using independent variables (treatment, dose, viral load, or CT). After analyzing the data distribution, analysis of variance and Kruskal–Wallis tests were performed for multiple groups. The analysis was performed using the R Studio statistical package (v. 4.3.1). The graphics were constructed using the GraphPad Software v. 8.0 (San Diego, CA, USA). The significance values were established as * *p* < 0.05, ** *p* < 0.005.

## 3. Results

### 3.1. HSV-1 Viral Load Kinetics in CNS

**Figure 2:**
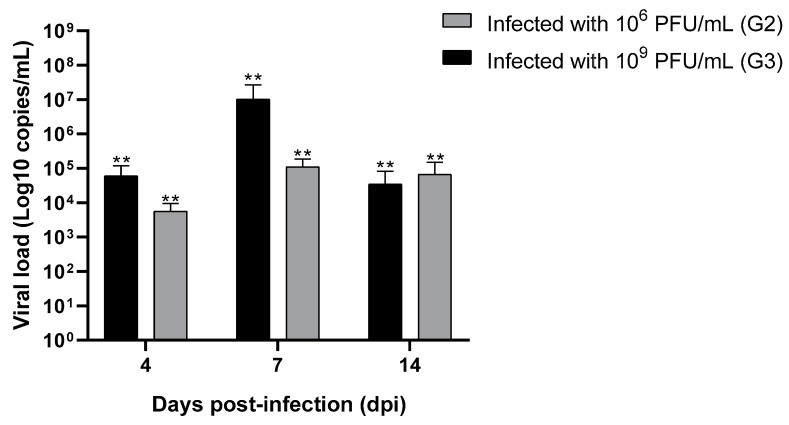
Mean viral load and standard deviation (SD) of the animals inoculated with 10^6^ PFU/mL (G2) and 10^9^ PFU/mL (G3) over time. The difference between viral load values at the three time points in group G2 (*p*-value < 0.001) and G3 (*p*-value = 0.002) was significant. ** Illustrates significant differences; *p* < 0.005. Error bars represent the SD.

Viral concentrations of 10^6^ and 10^9^ PFU/mL were tested to establish an infectious dose capable of safely reaching the CNS of BALB/c mice. All groups showed a high viral load in the brains of the animals. However, differences were observed between the viral loads achieved in the groups inoculated with 10^6^ and 10^9^ PFU/mL at 7 dpi (Figure 2). The animals in the G3 group had a high viral load of 1.0 × 10^7^ DNA copies/mL, and group G2 reached a mean viral load of 1.1 × 10^5^ DNA copies/mL. Moreover, animals infected with 10^9^ PFU/mL had an average viral load of 6.0 × 10^4^ DNA copies/mL and 3.4 × 10^4^ DNA copies/mL in the brain on the 4th and 14th dpi. In comparison, the G2 group had average viral loads of 5.5 × 10^3^ DNA and 6.6 × 10^4^ DNA copies/mL on the same days post-infection.

A higher viral concentration (10^9^ PFU/mL) at inoculation guaranteed a higher viral load on the seventh dpi; however, animals infected with log 10^9^ and 10^6^ PFU/mL had similar viral loads after 4 and 14 days. This result indicates that, regardless of the initial viral load, the HSV-1 viral load in the brain decreases after 2 weeks, which is consistent with the onset of viral latency. Therefore, the subsequent experiments were performed using a 10^9^ PFU/mL inoculum of HSV-1.

### 3.2. Detection of HSV-1 in Brain Tissues After Treatment

On days 4, 7, and 14 post-infection, the untreated group (G3) exhibited mean viral loads of 7.82 × 10^4^, 1.89 × 10^8^, and 4.27 × 10^4^ DNA copies/mL, respectively. In comparison, the acyclovir-treated group (G5) had averages of 1.95 × 10^4^, 1.92 × 10^7^, and 3.0 × 10^4^ DNA copies/mL on the same days. Conversely, the CRISPR/Cas9 anti-UL39 treated group (G4) displayed average viral loads of 4.7 × 10^4^, 3.4 × 10^5^, and 3.9 × 10^4^ DNA copies/mL. Statistical significance was observed in the median viral load variation across the groups (*p* < 0.001) on days 4, 7, and 14. Treatment with acyclovir demonstrated a statistically significant reduction in the mean viral load (*p* = 0.02), as did the viral load reduction observed with CRISPR/Cas9 treatment on post-infection days (*p* = 0.04).

**Figure 3:**
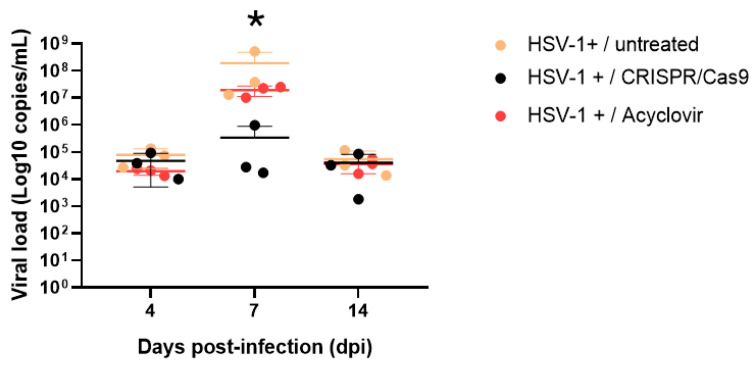
Treatment effect of CRISPR/Cas9 anti-UL39 on the HSV-1 viral load in the brain compared to the group untreated (G3) or treated with acyclovir (G5) on the fourth, seventh, and fourteenth dpi according to qPCR. The circles represent the viral load of each animal; beige (G3); black (G4); pink (G5). Error bars represent the SD of each experimental group (n = 28; at least three replicates for each dpi); * = *p*-value = <0.01 in 7 dpi.

The average viral load of HSV-1 in the brain tissue of animals treated with CRISPR/Cas9 (G4) reached 3.4 × 10^5^ copies/mL. In contrast, animals in the non-treated (G3) and acyclovir-treated groups (G5) exhibited 1.89 × 10^8^ and 1.7 × 10^7^ DNA copies/mL, respectively, at 7 dpi. Therefore, the viral load of the animals treated with CRISPR/Cas9 was reduced by up to 2–3 logs compared to that of the other groups at 7 dpi (Figure 3).

### 3.3. Pharmacological Treatment and Brain Cell Damage

**Figure 4:**
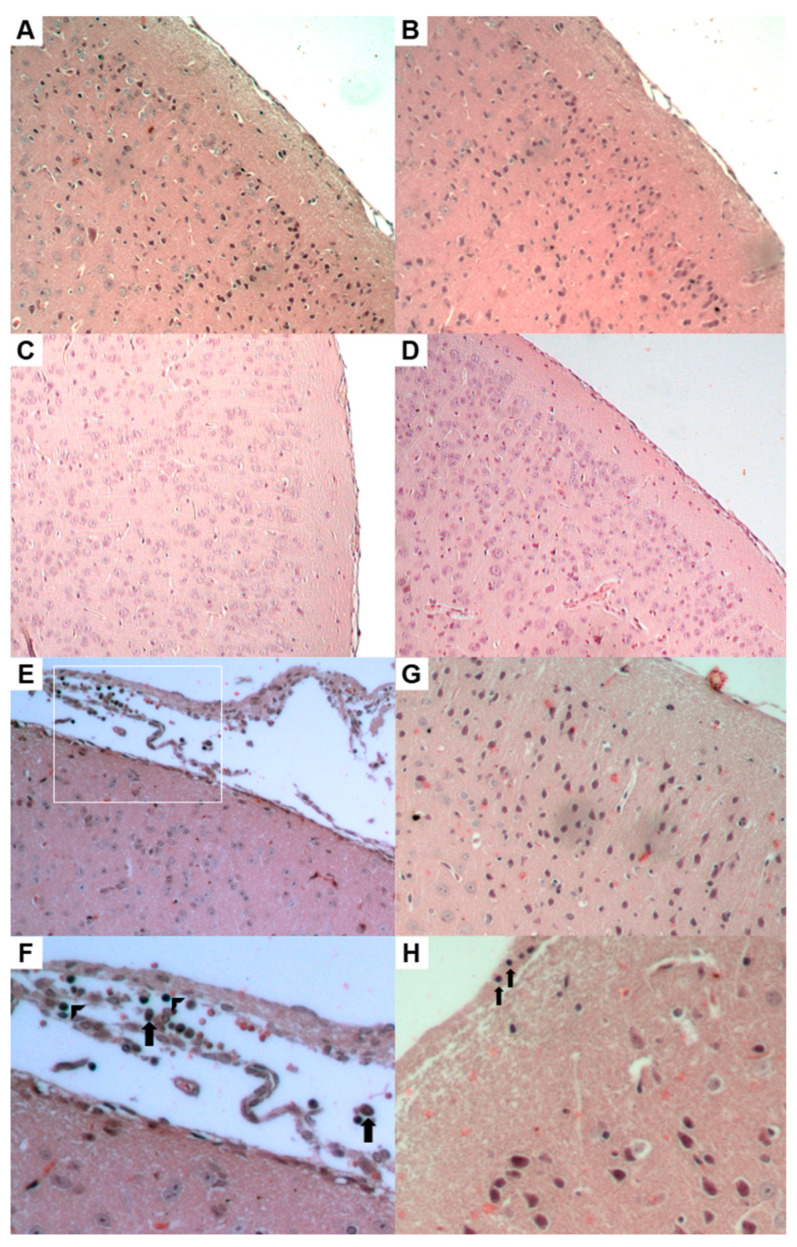
Hematoxylin and eosin (HE) staining of the cerebral cortex and meninges. (**A**) The cortex of the uninfected animals (control; G1). (**B**) The cortex of the uninfected and CRISPR/Cas9-treated animals (G6) that were treated under the same conditions. (**C**) The cortex of the infected and CRISPR/Cas9-treated animals (G4) at 7 dpi. (**D**) The cortex of the same group at 14 dpi. The histological sections of G1, G4, and G6 are without morphological changes or signs of inflammation or cell death. (**E**) The cortex of the infected and acyclovir-treated mice (G5) at 7 dpi; (**F**) arrowheads indicate lymphocytes, and arrows indicate plasma cells. (**G**,**H**) Cortex slides of the same group at 14 dpi; arrows indicate pycnotic nucleus. Each figure shows a representative sample of a group of five animals. (**A**–**E**,**G**) were captured at 200× magnification. (**F**,**H**) were captured at 400× magnification. The white box illustrates the magnification region of the figure (**F**).

The control group exhibited nervous tissue integrity, as indicated by the cortical samples (Figure 4A). No changes were observed in the histopathological analyses of CRISPR/Cas9 anti-HSV-1-treated mice that were not infected (Figure 4B). Similar morphological findings to the control groups, without notable changes to H and E, were observed in the HSV-1-infected mice and CRISPR/Cas9 anti-HSV-1 (G4) at 7 and 14 dpi (Figure 4C,D). Without chromatin compaction or inflammatory infiltration, these groups maintained the neuronal layers in the cortex and several other encephalic areas, such as the hippocampus and lateral ventricle (Figure 4A–D).

Although no inflammatory infiltration was observed in the cortical tissue (Figure 4E), an increased cellular population was observed in the meningeal tissue of HSV-1-infected mice treated with acyclovir (G5). Arrowheads indicate lymphocytes, and arrows indicate plasma cells (Figure 4F). The hippocampus and lateral ventricle were also assessed, and no findings were observed in the H and E-stained samples. Moreover, infected mice treated with acyclovir did not show inflammatory infiltration of the meningeal tissue at 14 dpi. However, pycnotic nuclei, which indicate cell death, were observed in the cortex at 14 dpi (Figure 4G,H).

### 3.4. CNS Infection with a Brazilian HSV-1 Strain Reaches Different Brain Regions

**Figure 5:**
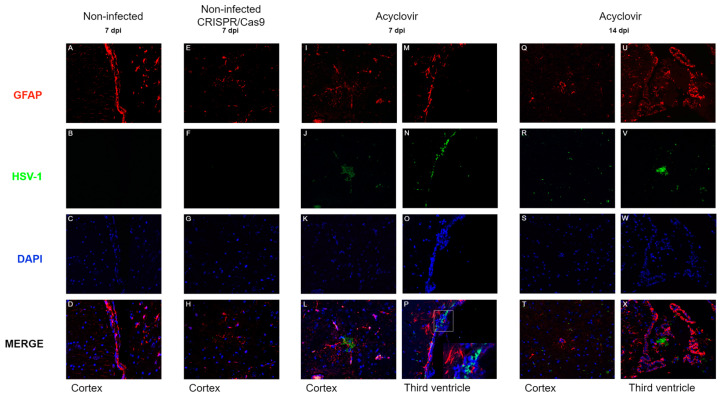
Immunofluorescence of brain tissue of non-infected and infected (10^9^ PFU/mL) mice. (**A**–**D**) The cortex of non-infected mice (control) at 7 dpi. (**E**–**H**) Cortex of non-infected mice treated with 200 ng CRISPR/Cas9 at 7 dpi. (**I**–**L**) The cortex of infected mice treated with 150 mg/kg acyclovir at 7 dpi. (**M**–**P**) Third ventricle of infected mice treated with 150 mg/kg acyclovir at 7 dpi; the detection of HSV-1 in a region populated by ependymal cells (**P**). (**Q**–**T**) The cortex of infected mice treated with 150 mg/kg acyclovir at 14 dpi. (**U**–**X**) The third ventricle of mice treated with 150 mg/kg acyclovir at 14 dpi. Co-immunofluorescent staining for HSV-1 antigens (green), GFAP-positive astrocytes (red), and nuclei (blue) were counterstained with 4,6-diamidino-2-phenylindole (DAPI). Images were captured at 400× magnification. The white box illustrates the magnification region of the figure (**P**).

The samples were stained for HSV-1 and GFAP, which are conventional astrocyte markers, to identify HSV-1 positivity. The non-infected control group and CRISPR-treated non-infected mice were positive only for astrocytes in all encephalic areas (Figure 5A–H). However, infected mice treated with acyclovir at 7 dpi were positive for the HSV-1 antigen in the cortex in the third ventricle (Figure 5I–P). Cortical samples showed disseminated HSV-1 positivity. However, GFAP-positive cells were consistently negative for the HSV-1 antigen. HSV1-positive cells (green) were observed in close proximity to ependymal cells; however, they were GFAP-negative in all samples. Mice infected with HSV-1 and treated with acyclovir after 14 days demonstrated HSV-1 antigens in the cortex and third ventricle (Figure 5Q–X) and as well as in the hippocampal area, in a layer compatible with hippocampal neurons (Figure S1).

## 4. Discussion

Infections caused by HSV-1 that affect the CNS are severe, and the patient is at risk of sequelae or permanent damage to nervous tissue [15,45]. This study used topical therapy (eye route) with CRISPR/Cas9 specific to the HSV-1 *UL39* gene in BALB/c mice infected with the Brazilian strain of HSV-1. The effects of CRISPR therapy on the CNS were evaluated. The findings suggest that CRISPR/Cas9 anti-UL39 therapy has beneficial effects, such as reducing the viral load in the CNS, and demonstrate the safety of the treatment, as indicated by the absence of neuronal cell damage based on histopathological findings.

Initially, the kinetics of HSV-1 infection in the CNS were evaluated. The kinetics observed in this study present a viral load greater than that observed by Meydin-Lamadé [46]. Their results, obtained from an infection of 10^5^ PFU HSV-1 intranasally, demonstrated viral loads in the CNS of 1.65 × 10^4^ and 5.34 × 10^4^ viral copies/μg DNA on the second and seventh dpi, respectively. The findings of this study are similar to those reported by Marques et al., who evaluated the viral load produced by intranasal infection with 10^6^ PFU/mouse HSV-1 in the cortex and subcortex. Their results showed viral loads >10^5^ PFU/g tissue between 5 and 7 dpi [47]. Viral inoculation via the ocular route has consolidated efficiency in producing neurological infections [48] and was effective in this study, even without corneal scarification, a standard method used for more than two decades [49,50,51]. These results suggest that novel experimental designs that preserve animal welfare should be used for CNS infection models.

Although gene editing mediated by CRISPR/Cas9 does not completely inhibit the virus from disseminating to the brains of treated animals, it can reduce the viral load. As demonstrated by the kinetics of the non-treated group, the viral load of HSV-1 in the CNS of mice was noticeable and high at 4 dpi and peaked at 7 dpi. Viral detection at the primary site of infection and in the CNS declines after 14 days when the early latent phase begins [22]. The kinetics observed in animals treated with CRISPR/Cas9 showed that the viral load or HSV-1 replication did not reach higher rates over time compared to untreated animals or those that were treated with acyclovir.

On the seventh dpi, the mean viral load reached 1.9 × 10^7^ DNA copies/mL in the acyclovir-treated group, whereas this dynamic was not observed in the CRISPR/Cas9-treated group (3.4 × 10^5^ DNA copies/mL). This finding demonstrates that the mechanism of action of the CRISPR/Cas9 complex in the eyeball can reduce the viral load in the CNS. These results indicate that the viral load did not decrease by the 14th dpi, suggesting that this treatment does not reduce the number of viral particles at the onset of latency in the CNS. However, a reduction in viral load was observed during the acute phase of the infection at 7 dpi. This reduction may be related to greater protection against cellular damage caused by the virus.

Histopathological analyses demonstrated the integrity of the nervous tissue of HSV-1-infected and non-infected animals that received a dose of CRISPR/Cas9. Ocular acyclovir treatment did not alter HSV-1 expression in the CNS. Treatment of CNS infections caused by HSV-1 involves the intravenous administration of high doses of acyclovir (30–60 mg/kg) and typically lasts for weeks [45]. However, this study only evaluated the application of acyclovir at a single dose via the ocular route because it is used for herpetic keratitis. Even at a high viral load, no inflammatory infiltrates were observed in the cortex, hippocampus, or lateral ventricle of group G5 on the 7th dpi. However, the meningeal tissue showed increased leukocytes on the 7th dpi, and pycnotic nuclei were observed on the 14th dpi, indicating cell death.

HSV-1 can reach different regions of the CNS through ocular infection, such as the cortex, cerebellum, hippocampus, brainstem, and olfactory bulb [22]. The innate immune response plays a crucial role in protecting the CNS against HSV-1 [52]. Microglia, astrocytes, oligodendrocytes, and neurons express receptors that recognize viruses and activate innate immune responses [53,54]. This study evaluated the presence of HSV-1 in astrocytes in the cortex, hippocampus, and third ventricle of animals treated with acyclovir to assess innate immune activity in the CNS. However, no HSV-1-positive astrocytes were detected in these regions. These results could be explained by the high selectivity of HSV-1 for neurons and epithelial cells. The region of the third ventricle, formed by ependymal cells of epithelial origin, was positive for the virus. Furthermore, the virus was detected in the cortical areas highly populated by neurons.

The CRISPR/Cas9 system efficiently reduces and inhibits the replication of herpes viruses in vitro. Van Diemen et al. reported that simultaneous targeting of essential genes with two or more gRNAs can inhibit the production of new HSV-1 particles, resulting in a decrease of >10^6^ DNA copies in viral titers in cell culture [55]. However, the results obtained using CRISPR/Cas9-mediated editing were not as impressive in in vivo models. When CRISPR/Cas9 anti-UL39 gene therapy was applied in vitro to an animal model, the inhibition of viral replication did not show a similarly high rate of decrease in HSV-1 CNS detection. However, this also occurred in a study performed by Aubert et al. using CRISPR/Cas9, which evaluated HSV-1-targeted endonuclease activity in mice with latent infection. However, < 4% editing was observed without detectable loss of the viral genome or therapeutic efficacy [56]. Recently, the same group used two meganucleases targeting the HSV-1 genome along with an improved delivery vector. This approach was remarkably more efficient, resulting in a reduction of HSV-1 genomes by over 90% in the superior cervical ganglion and over 50% in the trigeminal ganglion of treated animals [57]. These studies focused on interrupting viral latency in neuronal ganglia and are the only studies that have evaluated the efficacy of CRISPR therapy against HSV-1 in vivo.

Other groups assessed the effectiveness of CRISPR/Cas9 treatment in HSV-1-induced herpetic stromal keratitis (HSK). One in vivo study used the CRISPR/Cas9 system delivered via lentiviral particles as a preventive measure for HSK, which yielded promising results in reducing the viral load in the corneas. However, this study did not analyze the viral load in the brain [58]. The present study used a therapeutic approach shortly after ocular HSV-1 infection. It demonstrated that CRISPR/Cas9 treatment administered 24 h after ocular infection reduced the detected viral load in the brains of animals, even without using a delivery vehicle, such as viral vectors or nanoparticles. These findings also indicate decreased clinical signs of herpetic keratitis and diminished viral replication in the eyes of animals treated with CRISPR/Cas9 targeting *UL39* (unpublished data). However, CRISPR/Cas9-mediated treatment targeting the *UL39* mutation did not prevent the virus from disseminating to the CNS in the treated animals. Nonetheless, the average CNS viral load was reduced.

Recent studies on gene therapy for HSV-1 infection have emphasized the crucial role of CRISPR/Cas9 complex delivery methods in achieving higher efficacy in in vivo models [59]. While non-viral gene delivery vectors (e.g., lipid nanoparticles) can deliver therapy effectively [60,61], viral vectors remain advantageous because of their targeted cell- and tropism-specific delivery capabilities, minimizing off-target effects and ensuring therapy accumulation in desired tissues [62]. Additionally, the simultaneous use of multiple guide RNAs efficiently inhibits viral replication. The present study focused on targeting corneal and brain viral detection using an ocular treatment route. For neuroinfections, intravenous and intracranial routes are commonly used to enhance CNS delivery rates in antiviral and experimental gene therapies [63]. Despite the lack of statistical significance, the reduced viral load observed in the brains of animals treated with CRISPR/Cas9 targeting anti-UL39 underscores the promising results, encouraging further investigation with enhanced delivery methodologies.

Previously obtained in vitro data [31] show results of MTT cell viability assays revealed negligible decreases in the percentage of viable cells compared with controls at 24 h (0.5%) and 48 h (2.6%), indicating that the treatment with CRISPR/Cas9 anti-UL39 is relatively non-toxic. Although assessing animal welfare parameters reinforces the safety of treatment with CRISPR/Cas9 anti-UL39, histopathological data obtained from animals in groups G4 and G6 suggest the safety of CRISPR treatment. Topical ocular treatment with CRISPR/Cas9 offers advantages, for example, easy application, local action, and CNS protection, but intravenous acyclovir remains essential for neuroinfection due to its proven efficacy. The combination of both therapies could improve outcomes for HSV-1-related neuronal damage.

The limitations of this study include the small sample size (n = 8) for histopathological and immunofluorescence analyses, which did not include all groups (G1–G6) evaluated in the molecular tests. Unfortunately, two animals from group G2 removed the tag, and they were excluded from the study. In the group G6, only two animals were included due to the reduced number of animals available for the experiment. Furthermore, the short period of experimental infection evaluated did not allow the analysis of the interference of gene therapy in the latency period of the virus in the CNS. HSV-1 typically induces latent infections in its host that persist throughout its life with cycles of reactivation. This study only evaluated therapy over 2 weeks; therefore, whether the treatment had an impact on viral latency could not be investigated. Despite these limitations, this study highlights several aspects of in vivo infection by HSV-1 in the CNS based on ocular infection without corneal scarification, including DNA quantification in the brain after a high concentration of the viral inoculum, detection of the virus in specific regions of the CNS, and histopathological findings. These findings provide a basis for further experimental studies aimed at eliminating HSV-1 infections.

## 5. Conclusions

This study demonstrates that CRISPR/Cas9 anti-UL39 gene therapy can reduce the average viral load of HSV-1 in the brains of BALB/c mice treated with a single dose via ocular administration. The present study demonstrated viral detection in the brain peaked at 7 dpi. Topical CRISPR therapy did not adversely affect the integrity of the nervous tissue. Topical use of nucleoside analogues is common in herpetic infections; however, it does not reduce viral detection in the CNS. The findings of this study highlight the importance of investigating viral detection and evaluating antiviral treatments in the brain.

## Figures and Tables

**Table 1:** BALB/c mice infected or not with herpes simplex virus type 1 (HSV-1) and treated or not with CRISPR/Cas9 or acyclovir.

Experimental Groups		
G1	Non-infected (NI); negative control	n = 10
G2	Infected with 10^6^ PFU/mL	n = 8
G3	Infected with 10^9^ PFU/mL	n = 10
G4	Infected with 10^9^ PFU/mL and treated CRISPR/Cas9 (200 ng/μL)	n = 10
G5	Infected with 10^9^ PFU/mL and treated with acyclovir (150 mg/kg)	n = 10
G6	Non-infected and treated CRISPR/Cas9 (200 ng/μL)	n = 2

PFU, plaque-forming unit.

**Table 2:** Oligonucleotides used for the qPCR assay.

Oligonucleotides	Sequences
Primers	
HSV-1 UL-39 forward	5′-CCAACTGCACCATGATCATCGA-3′
HSV-1 UL-39 reverse	5′-GATGTTTGTCACCGCAACGAA-3′
Probe	
p HSV-1 UL-39	FAM-5′-CCCGCGCACCACGTC-3′-MGB

## Data Availability

The original contributions presented in the study are included in the article and Supplementary Materials, further inquiries can be directed to the corresponding author.

## Supplementary Materials

The following supporting information can be downloaded at: https://www.mdpi.com/article/10.3390/pathogens13121087/s1. Figure S1: Immunofluorescence of brain tissue infected (10^9^ PFU/mL) mice and treated with acyclovir (150 mg/kg) at 14th day post-infection.
