# Supplementary material for: CRISPR/Cas9 Eye Drop HSV-1 Treatment Reduces Brain Viral Load: A Novel Application to Prevent Neuronal Damage
Source: Pathogens. 2024 Dec 10;13(12):1087. doi: 10.3390/pathogens13121087 (PMC11676479; doi:10.3390/pathogens13121087)
Supplement: Supplementary file 1 [file pathogens-13-01087-s001.zip › pathogens-3346337-supplementary.pdf]

Supplementary file

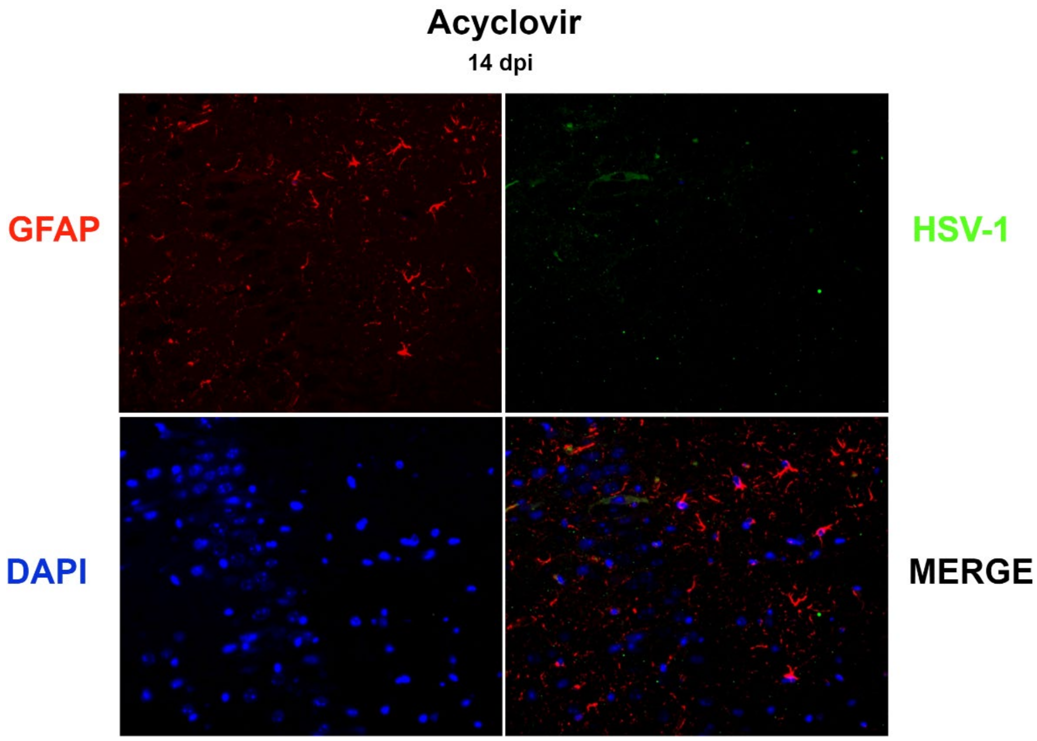

Figure S1. Immunefluorescence of brain tissue infected (10^9PFU/mL) mice and treated with acyclovir (150mg/kg) at 14th day post-infection. HSV-1 antigens were seen in regions compatible with hippocampus neurons. Co-immunofluorescent staining for HSV-1 antigens (green), GFAP+ astrocytes (red), and nuclei (blue) were counterstained with DAPI. Images were captured at 400x magnification.
